# High resolution non-invasive intraocular pressure monitoring by use of graphene woven fabrics on contact lens

**DOI:** 10.1038/s41378-019-0078-x

**Published:** 2019-08-26

**Authors:** Yushi Zhang, Yufeng Chen, Tianxing Man, Dong Huang, Xiao Li, Hongwei Zhu, Zhihong Li

**Affiliations:** 1 School of Information Engineering, Minzu University of China, 100081 Beijing, China; 2 National Key Laboratory of Science and Technology on Micro/Nano Fabrication, Institute of Microelectronics, Peking University, 100871 Beijing, China; 3 Key Laboratory for Advanced Manufacturing by Materials Processing Technology, Department of Mechanical Engineering, Tsinghua University, 100084 Beijing, China; 4 Center for Nano and Micro Mechanics (CNMM), Tsinghua University, 100084 Beijing, China

**Keywords:** Electrical and electronic engineering, Nanoscale materials

## Abstract

Monitoring intracorporal pressures are important for health care and diagnosis. In this work, a contact lens tonometer employing graphene woven fabrics (GWFs), which indicate great sensibility of resistance to strain, flexibility, stretchability, transparency, and biocompatibility, is proposed for real-time monitoring intraocular pressure (IOP) with high resolution. The mechanical properties of the device during the deformation were analyzed, and the sensitivity of the fabricated device was tested on a mimic human eyeball. In vitro experiments on porcine eyes were executed to test the effectiveness of the device. The change rate of resistance under different IOP was tested. Also, the relationship between the current changes and IOP variation when keeping the voltage constant for different devices was obtained. The contact lens tonometers with GWFs as high-resolution sensing element have shown a promising prospective to realize the low-cost disposable sensing contact lens with lower power.

## Flexible sensor: high-resolution, non-invasive intraocular pressure monitoring on contact lens

A high-resolution, non-invasive means for real-time monitoring of intraocular pressure has been developed using woven fabrics of graphene (hexagonal lattices of carbon atoms) on a contact lens. Monitoring intraocular pressure (IOP) is important for health care and diagnosis, such as with glaucoma. A Chinese team headed by Prof. Zhihong Li at Peking University and Yushi Zhang at Minzu University of China, cooperated with Prof. Hongwei Zhu at Tsinghua University, used graphene woven fabrics (GWFs) to measure IOP by contact lens. Tests under different IOPs demonstrated that the tonometer was able to accurately measure deformation of the eyeball caused by IOP. GWFs feature high transparency and biocompatibility, which makes them an attractive material as a sensor for tonometers. The authors believe their device offers excellent potential as a low-cost, disposable sensing contact lens with high resolution.

## Introduction

Human physiological index measurement has a significant role in biological research, medical detection, diagnosis, and therapy^1–3^. For example, intracorporal pressures, such as endocardial pressure, intracapsular pressure, intraocular pressure (IOP), and intracranial pressure, are important phenotypes for health care and diagnosis. Miniaturized devices based on micro/nanotechnology enable real-time monitoring intracorporal pressures. Nano-materials, such as graphene and carbon nanotube, can well fulfill the requirements of high sensitivity, flexibility, and biocompatibility for this kind of devices^4–6^.

In this work, we focus on measurement of IOP, which is a major feature to characterize glaucoma, a leading course of blindness^7^. The traditional devices, such as indentation tonometers and applanation tonometers, can be only performed in hospital and cannot monitor the IOP for a long time, which is necessary for some serious patients to be reminded for medical counter measure^7^.

The first miniature implanted tonometer for monitoring the IOP was reported in 1967 (ref. ^8^) and many follow-up tonometers have been developed since then^9,10^. Various new devices have been developed to monitor 24-h IOP^11–13^, but most of them are still implanted and have surgical risks. The contact lens tonometers were proposed and demonstrated several decades ago^14–16^. Recent years, the contact lens acting as a sensor carrier, has been studied widely in disease diagnosis with tests on tear glucose^17,18^, lactate^19^, etc. Coordinated with wireless display techniques, contact lens-based sensors have performed safe and convenient functions^20,21^. Thus, non-invasive tonometers using contact lens with sensing elements to monitor the IOP have been developed rapidly and already commercialized^22–24^. For example, the SENSIMED Triggerfish Sensor is a soft disposable contact lens embedding a telemetry chip and strain gauge sensor for continuous IOP monitoring^25–29^. However, the sensors on the contact lens are not sensitive enough, and therefore the complex and costly circuits are required to be built in the lens to amplify the signal and resist the noise. New sensing elements with high sensitivity can simplified the testing and realize the contact lens measurement with lower cost and power.

In our previous work, a novel material, the graphene woven fabric (GWF), which indicates great sensibility of resistance to strain, flexibility, and strechability, was developed^30,31^. As a stretchable sensor, the electrical resistance of GWF increases exponentially with tensile strain with gauge factors of ~10^3^ under 2–6% strains and ~10^6^ under higher strains that are the highest thus far reported, due to its woven mesh configuration and fracture behavior, making it an ideal structure for sensing tensile deformation by changes in strain^26^. Thanks to its transparency and biocompatibility, the GWF meets requirements as a strain sensor for tonometers. Because of the high sensitivity of the GWF, the tonometer has a promising prospective to realize a sensing contact lens with lower cost and power.

A simple contact lens tonometer with GWF as the sensing element was proposed and the preliminary results was reported in our previous paper as ref. ^32^, which enables monitoring IOP with high sensitivity. In this paper, the more technical details and latest results, including mechanical simulation and mechanical properties testing of the contact lens, are presented. The device was fabricated and the sensitivity was tested with the mimic human eyeball and the syringe pump. The current–voltage relationship of the device was recorded and the relationships between resistance change and deformation were calculated. The in vitro experiments on porcine eyes were executed and the change rate of the resistance under different IOP was tested. Also, the relationship between the current changes and IOP variation when keeping the voltage constant was obtained. The results illustrate that using GWFs as the sensing element on the contact lens can make the device more sensitive to the deformation.

## Materials and methods

### Principle and mechanical simulation

**Fig. 1:**
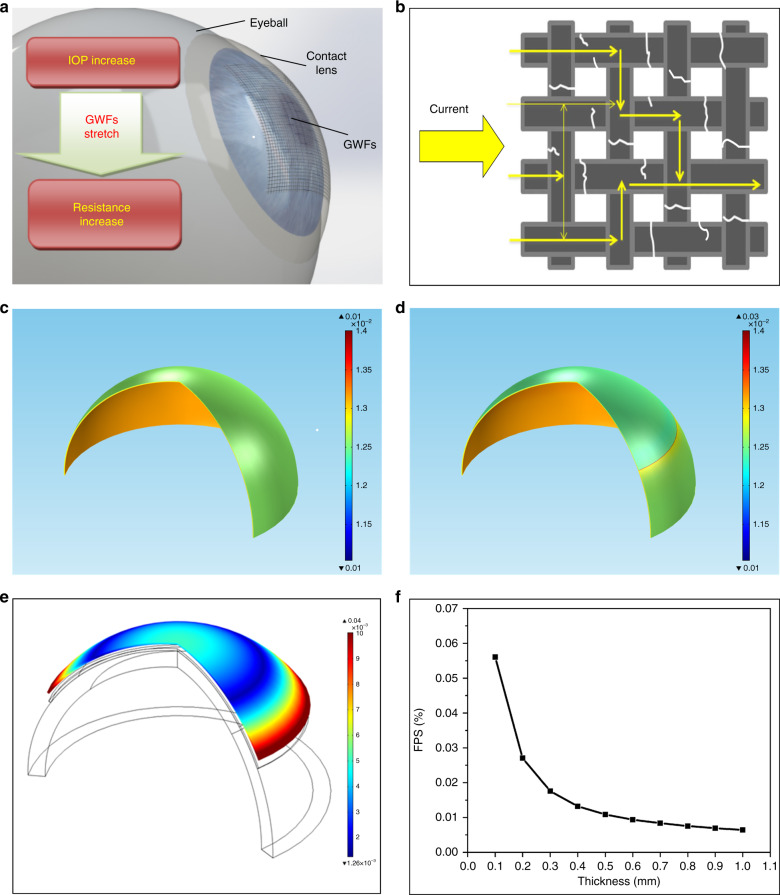
The principle and mechanical analysis of the device. **a** The working principle of the device; **b** current pathway through a fractured graphene woven fabric (GWF); **c** the first principal strains on the contact lens without GWF; **d** the first principal strains on the contact lens with the GWF; **e** the strains distribution of the contact lens in simulation; **f** the first principal strains of the cornea with different thickness

The working principle is schematically shown in Fig. 1a. A piece of GWF mounted on the outside surface of contact lens can be attached to the cornea tightly because of the homogeneous hydrophilicity. The small deformation of the eyeball makes the contact lens stretched as the IOP increasing, and therefore the piece of GWF stretches together. High-density cracks are appeared at weak point in GWF under small strains. With the strain increasing, the crack length and density gradually increase, which lead to the resistance of the GWF increasing, as shown in Fig. 1b. In a similar way, the recovery of IOP causes a decrease in resistance^31^. Thus, the IOP variation can be monitored by testing the current change under the constant voltage. As to the same piece of GWF on the flexible substrate, the formation of cracks in the GWF due to strain increase is repeatable and reversible, as reported in ref. ^31^. The specific electrical principle and resistance variation of the GWF stretching have been analyzed in ref. ^31^ and the mechanical properties of the device were studied when stretching in this work.

#### Interaction between GWF and contact lens

To understand the strain on contact lens contributed by GWF, simulation was performed with the solid mechanics module in the software of COMSOL. In the model, a thin film of GWF was attached on the outside surface of a hemispherical shell, with the thickness of 0.14 mm and radius of 8.6 mm. The Young’s modulus of the contact lens is defined as 1.5 MPa, whereas the one of the GWF is 0.15 MPa. The thickness of the GWF is defined as 10 nm. The outer surface of the hemispherical was defined as a free boundary and the internal pressures were added on the internal surface of the shell. The contact lens was stretched due to the strain caused by the increase of internal pressure. The strains of the contact lens both with the GWF and without GWF were simulated.

When the internal pressure is increased by 1000 Pa, the strain on the surface of the contact lens without the GWF is 1.257%, whereas the one with GWF is 1.255%, as shown in Fig. 1 c and d. This indicates that GWF contributes ignorable strain to the contact lens and can follow the deformation of the contact lens very well.

#### Interaction between device and eyeball

The IOP monitoring of the device was based on the deformation of the device. As the GWF contribute little acting force when stretching, the contact lens deformation can be approximately equal to that of the device with the deformation of the eyeball caused by IOP variation.

To research the mechanical properties, the strain of the contact lens during the deformation of the eyeball was simulated by the software of COMSOL. The contact lens model, with 8.6 mm base curve, 16.8 mm arc length and 0.14 mm thickness, was attached with the eyeball model. Increasing the inner pressure of the eyeball model to enlarge the eyeball, the contact lens deformed with the eyeball and the strains were calculated.

The model structure and the strain qualitative distribution were obtained as shown in Fig. 1d. Under tangential stretching and the radius of curvature increasing, the center and the edge of the contact lens have bigger strain than the points at other position. The strains at the center and the edge of the contact lens are about 0.4% and 0.8% individually under 20 mmHg IOP increasing. The curve indicates that the strain on the contact lens will decrease first and then increase as the distance, because of the out-of-plane strains decreasing and the tensile strain increasing from the center to the edge of the contact lens.

As the variation of IOP measurement is affected by corneal thickness, the relationship between the strain and the thickness of the cornea was studied by simulation. The first principal strain of the cornea under 1000 Pa variation with different thickness was shown on Fig. 1f. It shows that the strains of the cornea are different under the same IOP variation. Therefore, the devices should be calibrated by the Goldmann applanation method before used for each individual.

### Fabrication and experiments

#### Device fabrication

**Fig. 2:**
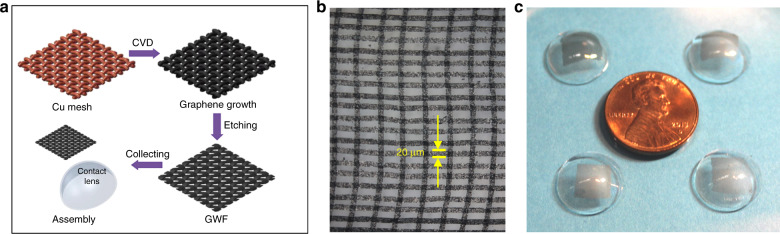
The schematic for fabrication and experiments. **a** The process of device fabrication; **b** the photo of the GWFs; **c** the photo of the device

The fabrication process of the device is schematically shown in Fig. 2a. A copper mesh was ultrasonically cleaned with hydrochloric acid and acetone to remove oxide and impurities on the surface. The treated copper mesh was used as the substrate of to grow GWF. In CVD process, the mesh was heated to 950 °C under 1000 mL/min argon gas flow and 60 mL/min hydrogen flow.

The temperature was maintained at 950 °C and argon gas flow was reduced to 200 mL/min. Hydrogen flow was cut off, while methane gas flow was introduced into the furnace at the rate of 15 mL/min for 20 min to grow multilayered graphene on the mesh. After cooled down to room temperature, GWF had the same planar structure as the mesh and was immersed into FeCl_3_/HCl mixture (1:1, mol/L) for 2 h to completely dissolve the copper. The finished GWFs were shown as the photo in Fig. 2b. After floating for 1 h in DI water to remove the remaining ions, GWF was collected by a contact lens (base curve = 8.6 mm, with 24% water content) and bonded on the outer surface of the lens as the water evaporated. The photo of the device was shown in Fig. 2c and copper foil tapes were attached to the GWF as electrodes in our first step testing.

#### Mechanical testing experiments

**Fig. 3:**
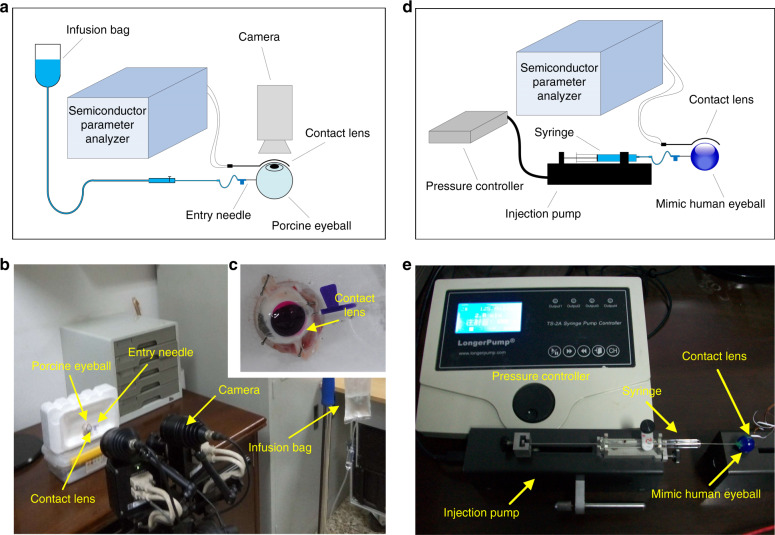
The setup of the experiments. **a** Setup for the mechanical testing and in vitro application experiments; **b** photo of the mechanical testing and in vitro application experiments setup; **c** contact lens attached with the porcine eye; **d** schematic for the sensitive performance testing; **e** photo of the sensitive performance testing setup^32^

To verify the simulation results, experiments were conducted to record the 3D major strain and displacement of real contact lens by the ARAMIS 3D System: the displacement was gotten by two cameras and calculated by the software. Contact lens was attached to a porcine eye connected with a transfusion bag to control the inner pressure by calibrating the height, as shown in Fig. 3a–c. Applying connecting vessels, we used the height of transfusion bottle to control IOP of porcine eye.

#### Sensitive performance testing

The fabricated device was attached to a mimic eyeball first for testing its sensitive performance under sphere deformation. The experimental setup was shown in Fig. 3d and the photo shown in Fig. 3e. With homogeneous hydrophilicity, the device could follow the deformation of the mimic eyeball well. A syringe pump was connected to the mimic eyeball to change the inner pressure of the mimic eyeball by injecting or extracting water. The contact lens was stretched with the mimic human eyeball dilated by the hydraulic pressure. According to the feature of GWFs, the resistance of the attached GWF was increased. The current–voltage relationship was tested under voltage sweep from 0 to 10 V with the curvature radius change of contact lens.

To further characterize the GWF properties as a sensing element during the sphere deformation, four devices were fabricated and labeled device 1 to device 4. To observe the relationship between GWF size and device performance, GWFs in each device had different side-lengths, which were 4, 5, 6, and 7 mm, respectively, shown in Table 1. Each device was attached to the mimic eyeball and a semiconductor parameter analyzer (HP 4156B) tested the electrical property of each device. When increasing the inner pressure by injecting water, the varied current was recorded with the voltage constant in 10 V.

**Table 1:** The average current rate of change in different range of the IOP for the two group devices^32^

The average current rate of change (%/mmHg)	The range of the effective IOP			
	0–5 mmHg	5–10 mmHg	10–15 mmHg	15–20 mmHg
Device number (side-length of GWF)				
Device 1 (4 mm)	14.7	3.89	1.98	0.69
Device 2 (5 mm)	23.6	3.68	2.28	1.02
Device 3 (6 mm)	23.6	3.83	2.51	0.98
Device 4 (7 mm)	16.7	4.20	1.87	1.04

#### In vitro application experiments

Three more tomometers with new improved GWFs were fabricated and attached to in vitro porcine eyeball to test its properties and performance for real applications. The setup of the experiment was shown in the Fig. 3a, which was the same as that in mechanical testing experiments shown in Fig. 3b. The contact lens was attached with the porcine eye, which was penetrated by the entry needle, shown in Fig. 3c. The contact lens was pink-dyed for better observation. A transfusion bag was connected to porcine to change the inner pressure by adjusting the height of the bag and the electrical testing were executed by HP 4156B semiconductor parameter analyzer. The tomometer could follow the deformation of porcine eyeball well and was stretched when the IOP increasing. To obtain the effectiveness and properties of the device, the resistance change rates of the devices under different IOP variation were tested and the varied currents when changing the IOP were recorded with the voltage constant in 10 V.

## Results and discussion

### Mechanical testing

**Fig. 4:**
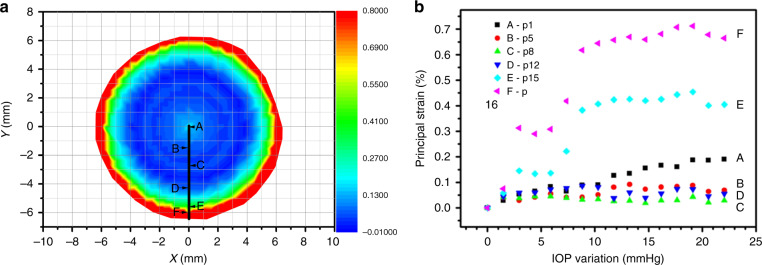
The strains during deformation by mechanical testing. **a** The strains distribution; **b** the strains variation with the intraocular pressure (IOP)

As the porcine eye dilated with increasing IOP, the contact lens was stretched and the major strain is qualitatively indicated in Fig. 4a. The major strain corresponds to the simulation result in distribution pattern. Points A to E were selected as shown in Fig. 4a and their strains in different effective IOP were shown in Fig. 4b. It shows increasing strains as the eyeball radium increasing. It also indicates that the strains on the contact lens decrease and then increase as the distance to the center increasing.

Compared with the strains of the contact lens under different IOP in the simulation shown in Fig. 1d, the strains at the center and the edge of the contact lens are about 0.2 and 0.7% individually under 20 mmHg IOP increasing, while these in the simulation are 0.4 and 0.8% individually. The quantitative difference between the simulation and the experiments are caused by the little parameter deviation in the simulation model to the non-ideal condition in mechanical contact, but the variation tendency show the consistency, which indicate the strains variation in the application of the device.

### Sensitive performance testing

**Fig. 5:**
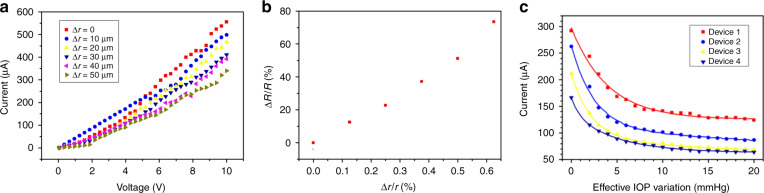
The results of sensitive performance testing^32^. **a** Current–voltage relationship of the device; **b** the device resistance change in percent (Δ*R*/*R*) to the change in percent of sphere’s curvature radius (Δ*r*); **c** the relationship between the current and IOP increasing under 10 V of the four devices

Tested by voltage sweep, the current–voltage relationship of the device in different curvature radius change was shown in Fig. 5a^32^. The deformation in different injection volume can be calculated and the relationships between resistance change and deformation were shown in Fig. 5b^32^, taking the near linear region of 6–10 V. The results show that using GWF as the sensitive element the device was more sensitive than the previously reported contact lens tonometers.

Moreover, the relationships between the current changes and effective IOP variation, shown in Fig. 5c^32^, can be obtained, according to the current testing results of the four devices, recorded by the semiconductor parameter analyzer. The average current rates of change in different ranges of effective IOP increasing for the devices were calculated and shown in Table 1^32^. It shows that the devices were far more sensitive to IOP variation when <5 mmHg. The average current rate of the four devices is 19.7%/mmHg in the range of 0–5 mmHg, in which the most sensitive one is 23.6%/mmHg, and 3.90%/mmHg in the range of 5–10 mmHg. For tonometer application, the sensitivity is highly demanded especially in small variation and 0–10 mmHg range is enough.

### In vitro application experiments

**Fig. 6:**
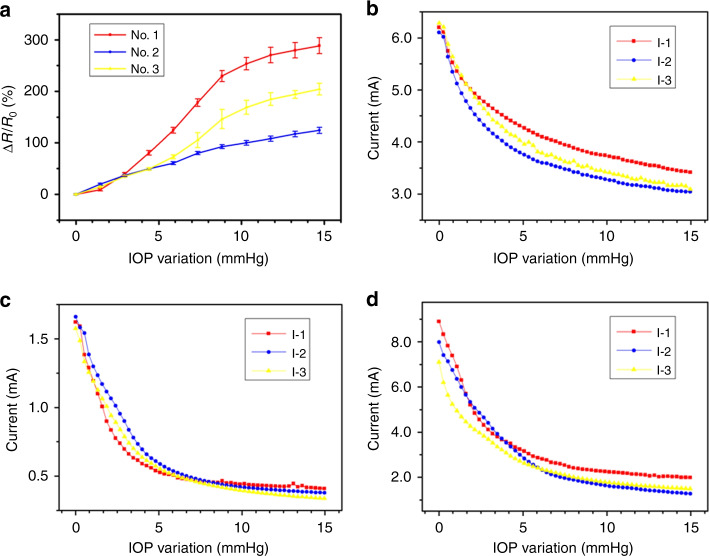
The results of in vitro application experiments. **a** The relationship between the resistance change rate and the IOP variation; **b**–**d** the relationship between the IOP variation and the current when keeping the voltage constant in 10 V

The relationship of three devices between the IOP variation and resistance change rate was shown in Fig. 6a. It shows that the device resistance change obviously with the IOP variation in 0–15 mmHg. The relationships between the current changes and IOP variation of the three devices, shown in Fig. 6b–d, can be obtained, according to the current testing results.

The average resolution is 6.8%/mmHg and the highest one is 7.3%/mmHg in the variation range of 0–10 mmHg, while it can be calculated the resolution is about 0.6%/mmHg in the pre-commercial research in ref. ^24^, which is effective enough for testing. It demonstrates that our tonometer can be sensitive and work well for testing the current with constant voltage, especially in the range of 0–10 mmHg variation.

Because of the subtle difference of the GWF piece individual, each device has different electrical property on the numerical, but the variation trends are uniform. The variation of IOP, which is affected by factors such as the corneal thickness and Young’s modulus, deduced from the strain measurement is different for individuals. Therefore, the device used for each individual should be calibrated by the Goldmann applanation method^7^, which is the gold standard for IOP measurement, before using for real-time monitoring. Tested and calibrated before using on the eye, the device can work well in high resolution. As the temperature approximately keeps constant on the human eyeball, its effect is on the IOP measurement not characterized in this work.

In the future work, when using in the “in vivo” human test, the coil will be integrated on the contact lens and the strain measurement can be read out wirelessly. As the repeatability and consistency for different pieces of GWF is not so good, each device should be calibrated before using for measurement. In addition, the packaging of the device has yet to be further studied for long-term reliability and stability.

## Conclusion

The deformation of the eyeball caused by IOP can make the strain of the contact lens. As the high sensitivity to the deformation properties of GWF, the resistance of the device can change significantly with the deformation of the eyeball. Increasing of the IOP can be monitored by the current changes when keeping the voltage constant, which can remind patients for medical counter measure. The average resolution of the device is 6.8%/mmHg in the variation range of 0–10 mmHg, which is much higher than the research before.

Besides, the transparency of GWFs is more than 80% and that of contact lens substrate is about 95%. Combining the highly strain sensing sensitivity and transparency, contact lens tonometers with GWF as high-resolution sensing element have a promising prospective to realize the low-cost disposable sensing contact lens with lower power.
